# Immunogenicity Associated with Botulinum Toxin Treatment

**DOI:** 10.3390/toxins11090491

**Published:** 2019-08-26

**Authors:** Steven Bellows, Joseph Jankovic

**Affiliations:** Parkinson’s Disease Center and Movement Disorders Clinic, Department of Neurology, Baylor College of Medicine, Houston, TX 77030, USA

**Keywords:** botulinum toxin, immunogenicity, immunoresistance, bioassays, clinical resistance testing, neutralizing antibodies

## Abstract

Botulinum toxin (BoNT) has been used for the treatment of a variety of neurologic, medical and cosmetic conditions. Two serotypes, type A (BoNT-A) and type B (BoNT-B), are currently in clinical use. While considered safe and effective, their use has been rarely complicated by the development of antibodies that reduce or negate their therapeutic effect. The presence of antibodies has been attributed to shorter dosing intervals (and booster injections), higher doses per injection cycle, and higher amounts of antigenic protein. Other factors contributing to the immunogenicity of BoNT include properties of each serotype, such as formulation, manufacturing, and storage of the toxin. Some newer formulations with purified core neurotoxin devoid of accessory proteins may have lower overall immunogenicity. Several assays are available for the detection of antibodies, including both structural assays such as ELISA and mouse-based bioassays, but there is no consistent correlation between these antibodies and clinical response. Prevention and treatment of antibody-associated non-responsiveness is challenging and primarily involves the use of less immunogenic formulations of BoNT, waiting for the spontaneous disappearance of the neutralizing antibody, and switching to an immunologically alternate type of BoNT.

## 1. Introduction

Botulinum toxin (BoNT) is a potent toxin that has proven remarkably useful in the treatment of a variety of neurologic and non-neurologic conditions [1]. Ever since the first study demonstrating its efficacy in blepharospasm [2], which eventually led to the approval by the US Food and Drug administration as the initial therapeutic indication for BoNT (along with strabismus and other facial spasms), the use of BoNT has expanded to multiple other indications and has provided therapeutic efficacy not possible with oral medications. However, there are some patients who lose their response to BoNT injections for a variety of reasons, including the emergence of immunoresistance as a result of formation of blocking or neutralizing antibodies (NAbs). In this review, we discuss the mechanisms and prevalence of antibody formation across BoNT therapy indications and BoNT formulations, as well as methods of detection of these antibodies and their relationship to clinical outcomes.

## 2. Definitions

Terminology used to describe BoNT outcomes is often confusing and not well defined. Primary non-response (PNR) refers to a situation in which patients in whom BoNT fails to improve symptoms from the very first injection and all subsequent treatments [3]. Secondary non-response (SNR) occurs when patients derive benefit from at least one injection, but lose that benefit over subsequent injection cycles. Loss of response can be either partial or complete. There are many reasons why initial responders lose their response; development of NAbs, i.e., immunoresistance, is only one of them (see discussion below).

### 2.1. Primary Non-Response

Several studies have reported that some patients have never responded to BoNT; hence, they were categorized as primary non-responders. For instance, in one series of 235 patients receiving BoNT for multiple indications, 9.1% of them were thought to have “primary resistance” and 7.5% “secondary resistance” [4]. Primary resistance, or PNR, was defined as a <25% response from the first injection despite two to three consecutive injections of increasing dosages. The etiology of the PNR was not discussed. PNR, however, is rare in clinic populations [5], and may be explained by low dosage, wrong muscles injected, presence of contractures, or prior vaccination against BoNT. NAbs can be present in individuals with previous vaccinations against BoNT. A pentavalent vaccination (against serotypes A–E) was used in approximately 8000 US military personnel during the Gulf War under an investigational new drug use [6]. BoNT-A titers, measured by mouse protection assay (MPA), were detectable in 28% of 169 soldiers who had received a vaccination 18–24 months prior to the testing. In 324 individuals who received a subsequent booster dose, antibodies were detectable in 99% after 24–36 days [6]. Titers potentially may also remain elevated in patients with previous botulism exposure [7]. BoNT potency may also degrade during storage [3]. The onabotulinumtoxinA (Botox^®^) package insert recommends storage of the 100 unit vials up to 36 months at 2 °C to 8 °C [8]. Higher temperatures of storage can increase degradation: RimabotulinumtoxinB (Myobloc^®^ in the USA or Neurobloc^®^ elsewhere) begins to lose potency at 9 months at 25 °C versus 30 months at 2 °C to 8 °C [9]. When reconstituted and refrigerated, potency loss begins at 12 h, and results in a 69.8% potency loss by 2 weeks [10].

It is also important to note that the diagnosis of BoNT insensitivity may be incorrect. For example, mistaking eyelid opening apraxia for blepharospasm [3] or pseudodystonia for dystonia [11] can lead to a wrong interpretation of poor or no response as BoNT insensitivity. Furthermore, some clinical conditions are notably difficult to treat with BoNT, such as anterocollis in cervical dystonia (CD), which may be wrongly interpreted as “unresponsive” to BoNT [3]. These pseudo non-responsive disorders are more prevalent than true immune-mediated primary non-responsiveness.

Due to some heterogeneity in response to BoNT some have proposed the possibility of genetic predisposition to non-responsiveness. One study assessed a genome database in search of mutations in genes coding for BoNT-A and BoNT-B protein binding sites and cleavage sites that may offer an explanation for primary resistance. However, mutations in the genes that code for pertinent residues in BoNT-A and BoNT-B binding and cleavage sites were either non-existent or extremely rare, strongly suggesting that patients generally do not carry genetic mutations that affect BoNT binding or cleaving activity [12]. Patients may have varied genetic predisposition to BoNT antibody formation due to different major histocompatibility complex (MHC). In one study of patients with spasticity and dystonia treated with BoNT-A, DQA1*01:02 and DQB1*06:04 were higher in NAb-positive than in NAb-negative patients [13]. In a study of BoNT-A light-chain and heavy-chain immunogenicity, various peptides (formed from segments comprising the total light or heavy chain) were evaluated using serum from NAb-SNR patients [14]. While some peptides were recognized by blocking antibodies of most patients, others were only recognized by a minority of patients, suggesting that some immune responses are under control of the patient’s MHC [14]. A minority of patients also demonstrate a variable immune response by developing flu-like symptoms and elevated cytokines with BoNT treatment. In one prospective study, 19 of 117 patients (16.3%) developed these symptoms and had significantly higher levels of IP10 cytokine [15].

### 2.2. Secondary Non-Response

Secondary non-response (SNR) is characterized by an initial benefit in symptoms after BoNT treatment followed at some point by a loss of response with subsequent injections. Most seasoned clinicians with long experience with BoNT have encountered patients who for some unknown reason fail to improve after a particular treatment visit even though they have consistently responded before and after that visit. Since these patients cannot be categorized as non-responders, we defined SNR as an absence of clinical response and absence of any adverse effects after at least two consecutive treatment visits [16].

As BoNT injections introduce a foreign protein capable of acting as an antigen, formation of antibodies has been a constant concern since the introduction of this therapy in the 1980s. Yet immunogenicity due to presence of NAbs is not the sole or even the main cause of SNR. Poor or no response to BoNT is more frequently due to an insufficient dose, inappropriate muscle selection, or improper injection technique or targeting [17]. In some patients natural progression of their underlying disease may account for the loss of benefit from BoNT therapy [3,18]. Other patients may have conditions that are known to be challenging to treat, such as pre-existing anterocollis (as noted above), or contractures as a result of long-standing abnormal posture. There can also be a discordance between patient and provider perceptions of benefit [17]. This has been borne out in multiple series involving patients with SNR, showing that only a portion of these patients have NAbs. In the portion of patients with SNR due to NAb, 81% begin initially with a partial loss of effect before progressing to complete a loss over an average of 2.5 injections [3]. In a meta-analysis of 8525 patients reported in 61 studies, the prevalence of NAbs was 3.5% among clinically responding patients and 53.5% in patients with SNR, but half of patients with SNR did not have NAbs [19].

The various studies, however, used different assays for NAbs, which accounts for the marked variability in the reported frequency of the NAbs and the poor correlation between the presence of NAbs and clinical response. In a series of 503 patients with SNR, only 44.5% of them were found to have NAbs by mouse hemidiaphragm assay (MHDA), discussed later in further detail [20]. Risk factors for development of NAbs include large doses per injection and large cumulative doses, certain formulations of BoNT (e.g., rimabotulinumtoxinB), and, most importantly, shorter injection intervals, particularly “booster” injections 1–2 weeks after a treatment visit [7,16,21,22]. Multiple studies have reported higher rates of immunogenicity in conditions requiring larger therapeutic doses of BoNT, such as CD [20]. In a study of 616 patients receiving BoNT for CD, Kessler et al. reported 17 with SNR, nine of whom were found to have NAbs [23]. Those with NAbs received significantly higher doses per treatment session, had shorter treatment intervals, and received more “booster” injections (reinjection within 6 weeks) than patients who continued to respond. In 22 patients with CD or oromandibular dystonia who developed SNR and were found to have NAbs, the mean dose per visit and total cumulative dose of BoNT were significantly larger than those without NAbs [16]. Since “booster” injections and shorter inter-treatment intervals have been associated with increased rates of antibody formation [7,20], longer inter-injection intervals (typically 12 weeks or longer) have been recommended and integrated into package inserts of the various brands of BoNT [7,24].

Despite a clear inverse relationship between inter-dose interval and risk of NAbs, there is growing interest in shorter injection intervals. This is largely driven by patient observations that benefits of injections frequently last less than 3 months and, therefore, many patients would prefer shorter inter-dose intervals to maintain efficacy. There is also a growing demand for the development of BoNT preparations that have a lower risk of immunogenicity [25]. One survey of patients noted peak dissatisfaction with BoNT injections just before their next injection when the beneficial effects of prior injection start wearing off [26]. More recent trials involving incobotulinumtoxinA have challenged the notion that shorter injection intervals are associated with a greater risk of SNR due to immunoresistance. In a randomized controlled trial assessing incobotulinumtoxinA for treatment of CD, 47.2% of patients had median injection intervals of less than 12 weeks, with no difference in the occurrence of new NAbs (detected by IPA and confirmed MHDA) throughout the 20-week study or 68-week extension [27]. Similarly, a trial of incobotulinumtoxinA in blepharospasm did not find any NAbs during the extension phase, despite half of the patients having median injection intervals of less than 12 weeks [28]. However, the mean dose per treatment visit, ranging from 64.7 units at the first visit to 72.7 units at the fifth visit [28], was substantially lower than the usual doses used in the treatment of CD or spasticity. In one study of 30 patients with a variety of dystonias who received shorter intervals of incobotulinumtoxinA dosing, with a mean 9.9-week inter-dose interval for a mean 14.3 injections, none of the patients demonstrated SNR [22]. These trials all used incobotulinumtoxinA, which may have lower rates of immunogenicity than other BoNT formulations (as discussed below).

## 3. Botulinum Toxin Structure and Function

BoNT is composed of a core neurotoxin and associated non-toxic accessory proteins (NAPs). The core neurotoxin consists of a 150 kD inactive precursor protein that contains a 100 kD heavy chain and 50 kD light chain, linked by a disulfide bond [29]. BoNT binds to glycoprotein acceptors on the cholinergic nerve terminal membrane, where native vesicle recycling mechanisms are used to facilitate uptake or endocytosis (BoNT-A via SV2, and BoNT-B via synaptotagmin) of the 50 kD light chain into the cytoplasm [30,31]. There, the light chain cleaves SNARE (soluble N-ethylmaleimide sensitive factor attachment protein receptor) proteins involved in eventual transport of acetylcholine (Ach) vesicles and their docking with the presynaptic membrane before releasing Ach into the synaptic cleft. Different serotypes of BoNT affect different SNARE proteins: SNAP 25 is cleaved by BoNT-A, BoNT-C, and BoNT-E, and VAMP (or synaptobrevin) is cleaved by BoNT-B, BoNT-D, and BoNT-F [24,29]. The impaired Ach exocytosis interferes with synaptic neural transmission in striate muscles as well as other cholinergically innervated structures, such as smooth muscles or exocrine glands, including salivary glands [24].

The core neurotoxin is typically accompanied by NAPs, comprised of hemagglutinin (50 kD) and non-hemagglutinin proteins (130 kD), that associate with the core neurotoxin to help prevent degradation [7,24,32]. Different BoNT types have different NAP compositions (including between preparations of the same serotype) [24]. IncobotulinumtoxinA is the only product where all NAPs have been removed during manufacturing [33]. These associations of core neurotoxin and NAPs are mixed with excipients, which vary among different brands, but include albumin, sucrose, lactose, sodium chloride, and disodium succinate [24]. DaxibotulinumtoxinA, a new formulation currently in development has a unique peptide excipient (described further below) [34].

Currently commercially available BoNT products contain either BoNT-A or BoNT-B serotypes. BoNT-A drugs include onabotulinumtoxinA (Botox^®^), abobotulinumtoxinA (Dysport^®^), and incobotulinumtoxinA (Xeomin^®^), while the only BoNT-B drug commercially available is rimabotulinumtoxinB (Myobloc^®^ or NeuroBloc^®^). Activity of these drugs is assessed by lethality assay (LD_50_), measured in mouse units (MU): However, assays by manufactures differ and preclude direct comparisons of potency between brands [24]. Although the individual units are not interchangeable, generally, one unit of onabotulinumtoxinA is equivalent to one unit of incobotulinumtoxinA [35], 2.5 units of abobotulinumtoxinA [36], and 50 units of rimabotulinumtoxinB [37]. Overall, BoNT-A has greater motor effects while BoNT-B has greater autonomic effects associated, for example, with dryness of the mouth due to reduced salivary secretions [24,38]. DaxibotulinumtoxinA is composed of a purified core neurotoxin without any NAPs (similar to incobotulinumtoxinA) and a stabilizing excipient peptide that binds to the neurotoxin [34]. This peptide is presumably designed to reduce diffusion of the neurotoxin and consequently increase its duration of effect. A phase 2 open-label trial of daxibotulinumtoxinA in 33 patients with CD noted a mean duration of effect of 25.3 weeks, longer than typical effect durations of other BoNT-A formulations [34]. A similar prolonged effect was noted in another phase 2 trial examining its use in the treatment of glabellar lines [39]. These findings, however, must be confirmed by larger, phase 3 studies that are currently being conducted in multiple centers.

## 4. Botulinum Toxin Antibodies

Antibodies against BoNT can be broadly divided into NAbs, targeting the core neurotoxin, particularly the binding site on the heavy chain, and non-neutralizing antibodies, typically targeting accessory proteins or clinically irrelevant sites on the core neurotoxin and which do not affect clinical efficacy. However, some antibodies have been found to bind to regions on the light chain of BoNT-A [40]. While these antibodies may also affect binding of the heavy/light chain complex, they may also impair downstream binding of the light chain to its target SNAP-25 substrate [14].

NAbs can decrease over a prolonged period of time. In one study we showed that the average duration between the detection of NAb and subsequent reversal to NAb negative status was 30 months (range, 10–78 months) [41]. In another series, 60% of patients’ titers eventually dropped after 6 years [42]. Unfortunately, the immunologic response to the same BoNT serotype can be reactivated by repeat treatments [41,43]. In some patients with partial SNR associated with NAbs, further increases in BoNT dosing can restore therapeutic benefit, but this may require up to four times the typical dosages of BoNT [44]. Plasmapheresis can remove NAbs and allow for resumption of therapy, but this is associated with increased risks and costs [3]. Intravenous immunoglobulin (IVIG) in doses up to 35 g has not been shown to be effective in reducing NAbs [45].

Antibodies directed against BoNT, typically of the IgG type, are sero-specific. Thus patients who fail to respond to BoNT-A because of the development of NAbs usually respond to BoNT-B. However, there is concern that those patients who switch serotypes are at a higher risk for developing resistance to the alternate type of BoNT. This is partly due to about 30% structural homology in the heavy chains of BoNT-A and BoNT-B [46]. Several studies have shown that patients who develop SNR to onabotulinumtoxinA may initially respond to rimabotulinumtoxinB but are likely to develop SNR to BoNT-B [3,21,43]. A similar risk is seen in studies switching from BoNT-A to BoNT-F [3,47].

Another strategy to regain clinical responsiveness in patients with SNR is to transition them to the formulation of BoNT that lack complexing proteins and, therefore, have potentially lower immunogenicity. Dressler et al. prospectively followed eight patients with CD and SNR associated with prior treatments with BoNT other than incobotulinumtoxinA [43]. After waiting for a complete drop in their BoNT-A NAbs titers, they resumed injections on these patients with incobotulinumtoxinA. All of these eight patients had regained the benefit of BoNT therapy, and no patients developed further SNR or another increase in BoNT-A titers over a mean follow-up of 1895.4 ± 1211.4 days [43]. While the small sample size may be a confounding factor, this study supports the potential use of incobotulinumtoxinA in patients with prior SNR to BoNT-A, after the NAbs have disappeared, and possibly its use as the initial formulation of BoNT.

Antibodies to the NAPs do not result in the loss of efficacy and are, therefore, not as clinically relevant as antibodies targeting the heavy or light chains [48]. Indeed, NAPs may serve to block access to antigenic sites on the core neurotoxin, and thus reduce immunogenicity [7]. One study examining antibody formation in response to vaccination with BoNT in rabbits showed higher antibody response to abobotulinumtoxinA compared to onabotulinumtoxinA, despite a lower NAP content in abobotulinumtoxinA [7]. This may be potentially explained by the inclusion of flagellin in abobotulinumtoxinA, a NAP that may serve as an adjuvant by interacting with toll-like receptors, involved in innate immunity that recognizes antigens with highly conserved molecular structures [49]. Additional studies [7,32,50] have suggested adjuvant effects from NAPs, but methodological concerns such as atypical preparation and excessive dosing amounts and frequency may have aberrantly increased immunogenicity [7]. However, this theory has driven development of products such as incobotulinumtoxinA and daxibotulinumtoxinA, where NAPs have been removed in an effort to reduce immunogenicity. This is supported by a relatively low frequency of antibody formation in patients treated with incobotulinumtoxinA [19,24,51].

Several factors relating to the structure of the BoNT itself, such as the ratio of active to inactive core neurotoxin, can affect its immunogenicity. Inactive protein refers to the 150 kD precursor protein formed prior to further protease cleaving into a 100 kD heavy chain and 50 kD light chain. Although inactive, this 150 kD protein bears the same epitopes as the heavy and light chain and remains immunogenic [52]. Consequently, products with a higher ratio of inactive to active toxin are more prone to generating antibodies and have a lower specific biological activity, which denotes the ratio of biologic activity to antigenic toxin [53].

Historically, the most notable example of product property affecting antigenicity is the transition from the original formulation of onabotulinumtoxinA to the newer formulation of onabotulinumtoxinA in 1997 [7]. The original formulation had higher amounts of inactive protein (up to 90%), with 25 ng of neurotoxin per 100 MU vial compared to 5 ng of neurotoxin in the newer preparation [20]. Antibody formation was found to be six times more likely with the original formulation than with the newer formulation [54]. BoNT-A is generally 95% activated during manufacturing, but approximately 25–30% of BoNT-B remains inactive, which may contribute to its antibody formation, discussed further below [55,56]. Variations in protein inactivation is not limited to different formulations: Changes in manufacturing process can lead to three-dimensional structure alterations, and degradation during storage can lead to unintentional inactivation of toxin [7,57].

Characteristics of the patients themselves may also affect rates of immunogenicity. Patients may already have antibodies related to prior botulism or vaccinations. Some have theorized that tetanus vaccinations may contribute to BoNT NAb formation, as tetanus toxin has a greater than 50% homology in amino acids to BoNT-A and BoNT-B. However, this has not been supported by animal data, and human data is not available [7].

## 5. Antibody Detection

When studying the effects of clinically relevant antibody-induced SNR, the ability to accurately detect and quantify antibodies becomes paramount. Various laboratory assays have been used to detect antibodies in patients with possible immunoresistance (Table 1). They can be broadly divided into structural assays and bioassays. Structural assays, such as ELISA and immunoprecipitation assays (IPA) [58], are sensitive in the detection of BoNT antibodies, but they are unable to discriminate between neutralizing and non-neutralizing antibodies. Bioassays such as the MPA or MHDA utilize animals to identify neutralizing antibodies that impact the clinical efficacy of the toxin.

The MPA has been historically considered the “gold standard” in the detection and quantitative measurement of BoNT NAbs [7,24]. In this assay, a patient’s serum is incubated with a standardized quantity of neurotoxin, and this neurotoxin is delivered via intraperitoneal injection into several mice. The results are based on the number of mice that survive due to the effects of NAbs in the patient’s serum. Some studies have used a qualitative version of this assay, where four mice are injected with the serum/neurotoxin combination, and a positive Ab results denotes the survival of ¾ mice [58]. Initial studies had a low sensitivity (47%) but high specificity (100%), with no false-positive results among MPA positive patients [59]. An additional study comparing the MPA to an IPA found similar sensitivity and specificity characteristics without false positives [58]. However, later studies have detected NAbs by MPA in rare patients who continue to respond clinically. In one study, four of 326 patients treated for their CD with onabotulinumtoxinA developed antibodies by MPA, but one of these four patients continued to have clinical responsiveness [18]. In another meta-analysis of patients treated with the lower protein formulation of onabotulinumtoxinA, 11/2240 (0.5%) across various indications developed NAbs by MPA [60]. However, seven of these 11 patients continued to have clinical response, but two of these seven had only a transiently positive test. Clinical responsiveness, however, is sometimes difficult to define, particularly when only one or two clinical evaluations are available, because of the placebo response and other factors. Although clinically useful, the MPA is not an ideal assay as it involves sacrificing animals, is very time-consuming, expensive, and semi-quantitative, and it requires a well-equipped and experienced laboratory [3,7]. The assay, although costly, is still commercially available at https://pacificbiolabs.com/?s=mouse+protection.

The MHDA utilizes ex vivo testing for NAbs. In this test, the left hemidiaphragm and phrenic nerve of a mouse are excised and placed in an organ bath. A serum/neurotoxin combination is applied to the assay, and the time to reach half-maximum of an electrically induced twitch force is measured. This measured time has a linear relationship to the amount of NAbs in the serum added to the assay [3,61]. The MHDA sensitivity has been reported at 0.0003 U/mL [20,62], or 25 times more sensitive than the MPA detection limit of 0.005–0.01 U/mL [63]. Given the higher sensitivity, fewer animals required, lower costs, decreased time to results, and less animal suffering, some have advocated for the replacement of the MPA by the MHDA [3,20,24]. However, the increased sensitivity has raised concerns for a higher false-positive rate (or at least detecting antibodies of uncertain clinical relevance), and thus difficulty in predicting SNR [7]. MHDA was used in the study by Albrecht et al., which reported 14% prevalence of NAbs among 596 patients treated with BoNT-A; 54% of whom were treated with abobotulinumtoxinA [51]. The authors, however, provided no data on the correlation between the presence of antibodies detected by the MHDA and clinical response. Although the assay is supposed to detect blocking antibodies, the authors noted that their patients were “still responding.” This observation strongly suggests that MPA is more clinically meaningful than MHDA, which may explain the differences in the reports of SNR among various studies. These findings underscore the importance of developing a standardized method for assaying NAbs that is both sensitive and specific for detecting clinically meaningful NAbs that are not animal-based, and that can be used to monitor BoNT-treated patients for the presence or emergence of immunoresistance.

Structural assays have largely been used as a screening test for NAbs, before confirmatory bioassay measurements are performed, due to their inability to discriminate between neutralizing and non-neutralizing antibodies [7]. In one of our early studies, designed to compare the Western blot assay (WBA) to MPA, relatively poor correlation was noted between the two assays with the WBA having low specificity [59]. Another assay, the IPA, measures the precipitation of radioiodinated BoNT (^125^I-BTX) when mixed with test sera [58]. One study compared IPA versus MPA in 83 patients (38 non-responders and 45 responders, mostly with CD). Sensitivities and specificities of these two assays were calculated using a patient’s level of clinical response, as well as their response to additional clinical tests such as the unilateral brow injection (UBI) and frontalis antibody test (FTAT), both described in the next section. All patients with a positive MPA result also had positive IPA results, but 19 patients with positive IPA results had negative MPA results. Furthermore, 14 of these 19 patients had SNR (two of whom later became positive by MPA), and five of the 19 continued to respond and were classified as false positives. IPA overall had a higher sensitivity (77–90%) versus MPA (30–50%) but a lower specificity (81–89%) versus MPA (100%), using clinical responsiveness, UBI, and FTAT as reference results [58].

ELISA tests have been used in the past, but without clear assessment of their quality [64]. A more recent study compared the specificity and sensitivity of ELISA versions of varying BNT-A1 coating antigen concentrations, using the results of the MHDA as a reference standard [64]. By adjusting the cut-off value of the positive result, they were able to calibrate the ELISA to maximum sensitivity (100% sensitivity and 55% specificity) or maximum specificity (100% specificity and 90% sensitivity) as compared to referenced results determined via MHDA testing. Varying sensitivities and specificities were noted with other antigen coating concentrations and serum dilutions. Similar to other studies, the value of the test seemed to lie in its sensitivity and screening potential, while bioassays such as MHDA and MPA are still required as confirmatory tests.

## 6. Clinical Resistance Tests

**Figure 1:**
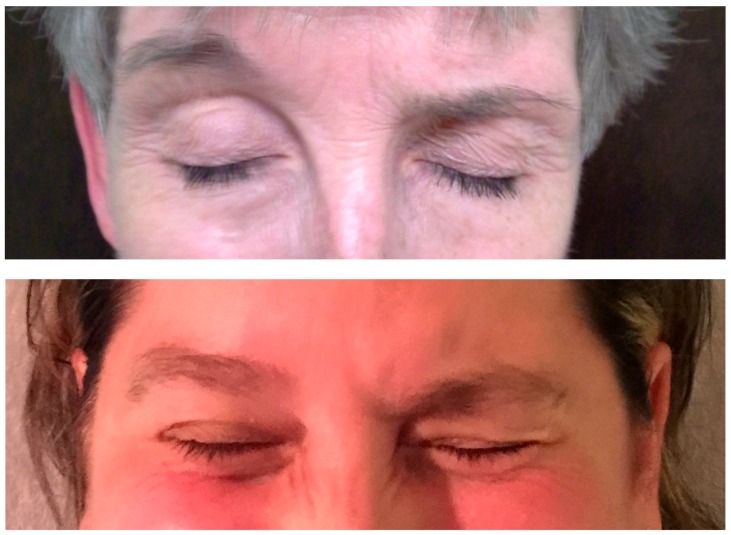
Unilateral brow injection (UBI) right medial eyebrow weakened by botulinium toxin (BoNT), hence the patient is responding (no immunoresistance). Legend: By convention the following dosages are injected into the right medial eyebrow: 20 U of onabotulinumtoxinA; incobotulinumtoxinA, 50 U of abobotulinumtoxinA, or 1000 U of rimabotulinumtoxinB. About 2 weeks after BoNT injection the patient is instructed to frown and describe whether the frown is symmetric or asymmetric and then send a picture of the upper face depicting the frown to the treating physician.

Given the difficulties of accessing reliable assays that do not require sacrificing animals and are relatively affordable, there has been growing interest in clinical tests of immunoresistance that are easy to perform and interpret and that correlate well with therapy response (Table 2). In this regard, we developed the UBI as an easy and reliable clinical method of screening for SNR. The UBI test consists of injecting a standard amount of BoNT in the right (by convention) medial eyebrow (Figure 1) [18]. The following dosages have been used traditionally as they are sufficient to paralyze the unilateral corrugator/procerus muscles: 20 U of ona- and incobotulinumtoxinA, 50 U abobotulinumtoxina, and 1000 U rimabotulinumtoxinB. After sufficient time for the BoNT to take effect (typically 1–3 weeks), the patient is instructed to frown and observe in the mirror whether the frown is symmetrical or asymmetrical [18]. The patient is also instructed to send us self-photographs showing normal facial expression and a frowning expression, which helps to document any asymmetry, indicative of absence of SNR (Figure 1). Since essentially all individuals normally frown symmetrically, asymmetric frowning is indicative of responsiveness to the injected BoNT that has weakened the right corrugator/procerus muscles. In contrast, symmetric frowning indicates that the injected muscles were not weakened and, therefore, the patient is likely immunoresistant to that type of BoNT. In the initial study comparing MPA to Western blot, several patients were also given UBI; eight patients who were SNR had concordant negative UBI results (no asymmetry was noted), and five of six patients who were clinical responders had positive UBI results (asymmetric frowning). The one patient with discrepant results was noted to have a borderline clinical response. Although it was used in only a portion of the patients, the sensitivity of the UBI was higher than the MPA in this study [59]. In another study comparing MPA to IPA in antibody detection, 29 patients received UBI in addition to undergoing these assays. Nine of the 10 patients with SNR had negative UBI results (with the discordant patient being –MPA but +IPA); 15 of the 19 patients with clinical response had positive (asymmetric) UBI results; three of these four discordant patients had borderline clinical response, which was reduced from their initial response to BoNT-A [58]. Over the past 3–4 decades, we have used the UBI and have found the test to be reliable in detecting SNR.

The rationale behind the frontalis antibody test (FTAT), used in the past by some investigators, is similar to the UBI, but involves injection of BoNT into the frontalis muscle and subsequent assessment for asymmetry of forehead wrinkling on eyebrow elevation [59]. Since the unilateral weakness of the injected frontalis muscles results in asymmetric elimination of wrinkles, often associated with the lowering of the ipsilateral eyebrow, the FTAT is not cosmetically acceptable and, therefore, we prefer UBI to FTAT. In the same study comparing antibody detection by MPA to Western blot, 10 of 11 patients with SNR who underwent FTAT had concordant negative results; one patient with clinical response underwent FTAT and had a concordant positive (asymmetric) result [59]. In the study comparing MPA and IPA antibody detection, 19 patients underwent FTAT: 10 of 12 patients with SNR had concordant negative (symmetric) FTAT results, and seven of seven patients with clinical responsiveness had concordant positive (asymmetric) FTAT results [58]. The two SNR patients with discordant FTAT results were both negative MPA, but one had a positive IPA result.

In the extensor digitorum brevis (EDB) test, the EDB muscle compound muscle action potential (cMAP) is measured by electromyography (EMG), followed by injection with a test dose of BoNT-A [65]. A positive response is defined as less than 50% decrease in cMAP amplitude. Initial studies noted an 80% sensitivity and 94% specificity with EDB testing for SNR to BoNT-A [65]. In one study, 20 patients with CD and SNR to BoNT-A were assessed clinically by the Toronto Western Spasmodic Torticollis Rating Scale (TWSTRS), and the EDB test was performed before the first visit and 15–21 days after a second injection [66]. NAbs were assessed primarily by IPA in which a positive clinical response was defined as a >30% improvement in TWSTRS. In the 10 patients with negative IPA, there was a mean EDB decrement of 85%. In contrast, of the 10 patients with positive IPA, there was a wide range of cMAP decrement, but seven of the 10 patients had decrements of less than 23%. The remaining three patients with larger decrements were noted to have low NAbs titers by IPA. Clinically, 11 patients (two with negative IPA and nine with positive IPA) had sustained SNR after injection with an EMG-guided technique. Of these 11 patients, seven (all with positive IPA) had SNR by EDB testing. The other four patients with negative EDB results had low or no NAb titers by IPA [66]. EDB testing results thus overall correlated well with high NAb titers by IPA.

In the sternocleidomastoid (SCM) test, a patient’s SCM maximal contraction is measured via two surface electrodes [67]. The SCM is then given a test dose of BoNT, the maximal contraction is re-measured by surface electrodes 14–21 days later, and the reduction in maximal contraction is calculated as a percentage. Reduction percentages that are two standard deviations below a previously measured mean reduction in a control group are considered abnormal. In the initial study describing this procedure, 17 patients with both partial and complete SNR were assessed by MPA, MHDA, and SCM testing [67]. These three tests were nearly entirely consistent: Six patients had evidence of NAbs by MPA, MHDA, and SCM testing, and 10 patients had no NAbs by MPA, MHDA, and SCM testing. Only one patient with evidence of NAbs by SCM testing had no NAbs initially by bioassay: However, a subsequent MPA performed 18 months later returned positive [67]. SCM detection of NAbs was thus highly consistent with bioassay tests.

The ninhydrin sweat test (NST) examines the effect of a test dose of BoNT on sweat production. In this test, the patient is brought to a room with controlled temperature and humidity, and presses their palm on a paper, which is then dyed and fixed with a 1% ninhydrin solution. A test dose of BoNT-A is injected into the hypothenar eminence, the palm sweating is re-measured by the same procedure after three weeks, and the ninhydrin-stained sheets are compared to determine the area of anhidrosis [68]. In the initial study where 18 patients (17 with CD and one with foot dystonia) with SNR were tested by MHDA and NST, the mean anhidrotic areas were smaller in NAb-positive patients (43.4 mm) than in the NAb-negative patients (412.3 mm) [68]. The anhidrotic areas were significantly correlated with NAbs titers as measured by MHDA [68]. Another study looked at both NST and MHDA results in 14 patients with CD and continued clinical responsiveness. Only one of 14 patients had a positive MHDA titer, with concordant small anhidrotic area (0.26 cm^2^). Detectable titers (0–0.9 mU/mL) that still qualified as negative MHDA results were found in six of the 14 patients, and their anhidrotic areas were significantly smaller than both patients without any detectable NAb titers by MHDA and control patients. This data suggests that NST may be able to detect patients with subclinical resistance [69].

## 7. Immunogenicity by Indication

Given the variability in indications, dosing strategies, BoNT formulations, definitions of SNR, and even assays used to detect NAbs, gathering and comparing data on antibody formation is a challenging prospect. Nevertheless, some consistent patterns have emerged from the review of the literature.

As noted previously, higher doses of BoNT tend to increase the risk of SNR due to immunoresistance. In patients treated with the original onabotulinumtoxinA formulation for blepharospasm, those receiving <500 units/year had a 4% prevalence of antibodies, while those receiving >500 units/year had a 63% prevalence of antibodies [7]. Similarly, several studies have noted that indications with lower total required BoNT doses, such as hemifacial spasm (HFS), tend to have higher sustained responses than those with higher typical doses, such as CD. A 10-year retrospective review of 235 subjects treated with BoNT found the highest rates of sustained response (defined as >50% improvement from baseline) at 2 years in HFS (96%), followed by blepharospasm (92%) and CD (68%), with similar rates at 5 years [4]. In Albrecht et al.’s cross-sectional analysis of patients with NAbs, the highest prevalence was noted in CD (15.7%), followed by other dystonia conditions (17.3%), blepharospasm (5.6%), and HFS (0%) [51]. This supports a direct correlation between the dose of BoNT and the presence of NAbs.

Spasticity remains an outlier in the aforementioned pattern. While some studies have noted higher rates of NAbs formation in spasticity indications (such as in SNR populations [20]), rates of antibody formation seem to be lower than expected. In a meta-analysis including four studies examining onabotulinumtoxinA in spasticity, only 1/317 patients (0.3%) developed new NAbs (measured by MPA), despite the highest mean doses across indications [60]. In another study of 279 patients receiving long-term treatment of upper limb spasticity with onabotulinumtoxinA, only one of 224 tested patients (<0.5%) developed new NAbs by MPA associated with clinically reduced responsiveness [70]. In an open label trial of treatment of upper limb spasticity with abobotulinumtoxinA over three cycles, no antibodies were found by MPA in 41 patients [7,71]. In the TOWER study examining escalating doses of incobotulinumtoxinA (400, 600, and 600–800 U over three cycles) in 155 patients with spasticity, none of the patients developed NAbs as determined by MHDA [72].

The presence of NAbs has been found to be quite rare in other BoNT indications. Several studies have found low overall prevalence of NAb formation when BoNT was used to treat overactive or neurogenic bladder. In one study examining 180 patients who underwent one cycle of onabotulinumtoxinA, no NAbs were found by MPA [73]. In a meta-analysis examining BoNT-A immunogenicity, 22 patients with overactive bladder were treated with onabotulinumtoxinA, and none of the patients developed NAbs [60]. However, these studies typically evaluated patients after only a single treatment cycle, although antibodies have been found in association with treatment failure in this indication [74]. In another series, 25 patients were identified who had already been treated for overactive or neurogenic bladder with one to seven prior injections [75]. They received another injection (24 with onabotulinumtoxinA and one with incobotulinumtoxinA), and antibodies were assayed pre and post-injection by the MHDA. NAbs were found in eight of the 25 patients (16%); none of the patients with NAbs were noted to have a “very good” response, but the remaining five of eight patients with NAbs were satisfied with their outcome.

Cosmetic indications have been generally associated with low frequency of NAb formation, probably because of a relatively low dose requirement. In one study of 363 patients receiving four injections with onabotulinumtoxinA over 64 weeks for treatment of glabellar lines, no NAbs were detected (by unclear assays) [76]. According to the package insert for abobotulinumtoxinA, only three of 1554 (0.19%) subjects enrolled in a variety of phase III trials developed new binding antibodies [77]. None of these patients had antibodies by MPA, and none had SNR [7]. In subsequent analysis of pooled studies involving 1968 patients, no patients were found to have antibodies over 13 to 17 months of abobotulinumtoxinA injections [78,79]. Another phase III trial utilizing incobotulinumtoxinA injections in 105 patients over 84 days noted no new NAb development [80]. Other indications, such as for axillary hyperhidrosis, have similar low immunogenicity. In a 52-week open-label study of treating axillary hyperhidrosis with onabotulinumtoxinA in 141 patients, no new neutralizing antibodies were found [81].

## 8. Immunogenicity by Formulation

### 8.1. OnabotulinumtoxinA

The original onabotulinumtoxinA formulation used before 1997 has had resistance rates (Table 3) ranging from 5% to 17% (with the highest rate noted in a cohort of patients with CD) [18,54]. During the first descriptions of SNR with this formulation, NAb formation was initially estimated at 7.1% [25]. In another cohort of 76 patients receiving injections since 1988, eight patients (10.5%) were found to have SNR [25]. A 2017 meta-analysis of five previous studies noted an overall NAbs prevalence of 7.2% [82]. A retrospective analysis of 106 patients with CD noted an incidence of 1.7 cases of SNR per 100 person-years, but no antibody data was available [4]. One study examined patients who had received BoNT injections for longer than 12 years; 45 patients were included, and they had all received their first injection between 1985 and 1989, thus having at least some cycles with the original preparation of onabotulinumtoxinA. Indications were spread between CD (43%), cranial dystonia (26%), and blepharospasm (11%). Among the 45 patients, 22 (49%) were found to have SNR, and 16/22 (73%) improved with adjustment of doses and targeting selection. Of the six patients of 45 (13.3%) with sustained SNR, only four were found to have antibodies by MPA. Thus, SNR is not always associated with NAbs, suggesting reasons other than immunogenicity may be responsible for SNR.

As discussed earlier, the newer preparation of onabotulinumtoxinA, introduced in 1997, reduced the amount of inactive toxin, resulting in a marked decrease in immunogenicity [7,54]. These lower rates were further confirmed in a prospective multicenter study conducted in 2008 evaluating the immunogenicity of onabotulinumtoxinA in CD [18]. In this study 326 patients were evaluated, 85.9% of whom received five treatments, with a mean treatment duration of 2.5 years. Only four of 326 patients (1.2%) developed NAbs by MPA. Among the four MPA positive patients, one continued to have clinical responsiveness despite the presence of NAbs. In keeping with previous data regarding SNR, most patients with documented SNR did not have NAbs. After excluding 25 patients where SNR status had been misinterpreted (and patients had continued to have clinical response), 48 had documented SNR: 24 responded at a later visit, 17 of them discontinued the trial, and only seven of the patients were determined to have true SNR, including the three patients with positive MPA and SNR.

Additional literature has supported this low rate of NAb formation. In 2010, a meta-analysis examined NAb-formation rate with onabotulinumtoxinA use for varying indications over 16 studies [60]. Across all indications, 11 of 2240 subjects (0.5%) were found to have NAbs during treatment. Of these 11 patients, only four of them (0.2%) lost clinical responsiveness. In an additional three randomized controlled trials involving 496 patients no NAbs were detected [60]. Even indications with typically higher dosage requirements had low rates of antibody formation, such as in CD (1.2%) [18] or spasticity (1/317 patients, or 0.3%) [60]. Another more recent meta-analysis found an NAb prevalence of 3.6% in 552 patients who participated in six studies [82].

### 8.2. AbobotulinumtoxinA

Antibody formation rates range up to 3.6% per the package insert [77]. A recent meta-analysis found a NAb prevalence of 1.4% in 3326 patients over 14 studies [82]. Prior double-blind placebo controlled studies with subsequent open label extensions have noted NAb rates of 0.9% [83] and approximately 2% [86]. In another study of 357 patients with CD who had received at least six injections with abobotulinumtoxinA, 17 patients were determined to have SNR, only nine of whom were found to have NAbs (2.5%) [23]. A study of long-term treatment of abobotulinumtoxinA use in CD found a SNR rate of 3.3% in 90 patients who had received treatment for 10–12 years [87]. None of these patients were found to have NAbs by MHDA. As mentioned above, an open-label trial of abobotulinumtoxin in the treatment of upper limb spasticity found no NAb formation in 41 patients over three cycles [71]. We previously noted the 14% frequency of NAbs in the study by Albrecht and colleagues [51] in which majority of the 596 patients were treated with abobotulinumtoxinA. Although there are many limitations to this study, the relatively high frequency of NAbs may be due to the fact that abobotulinumtoxinA contains greater amounts of active neurotoxin than similar doses of other products [88].

### 8.3. RimabotulinumtoxinB

The rimabotulinumtoxinB package insert estimates NAb formation at 18 months at a prevalence of 18% [85]. Initial regulatory submissions to the FDA for rimabotulinumtoxinB reported a rate of 22.6% of NAbs formation at 610 days [89]. A meta-analysis of 61 studies looking at NAbs formation with BoNT therapy included 1257 patients treated with rimabotulinumtoxinB, and noted an overall NAbs prevalence of 42.4%, compared to frequencies of 0.5% to 2.5% in BoNT-A formulations [19]. Similarly high rates of NAbs have been noted in multiple studies examining rimabotulinumtoxinB use in CD. For example, in one trial 31 of 90 patients (34.4%) treated over 42 months developed de novo [56]. In four prospective multicenter trials conducted over 18 months, rates of de novo NAb ranged from 33% to 44% [89]. However, the clinical relevance of these antibodies was uncertain. Paradoxically, higher percentages of MPA positive compared to MPA negative patients remained in the study. Larger proportions of patients who withdrew due to a lack of efficacy were MPA negative. Higher doses overall were used in MPA positive patients, but they did not require an escalation of dose over the study. The authors noted that these higher doses did not suggest that MPA positive patients had decreased response to BoNT: Rather, it suggested that patients who required higher doses of BoNT were more prone to developing MPA positive results.

### 8.4. IncobotulinumtoxinA

IncobotulinumtoxinA, in contrast to other BoNT preparations, lacks NAP, which may explain its relatively low immunogenicity, perhaps by reducing a theoretical adjuvant effect. In the prior meta-analysis of 61 studies examining BoNT immunogenicity, incobotulinumtoxinA was found to have the lowest rate of NAb formation at 0.5% [19]. In an initial study examining incobotulinumtoxinA use in upper limb spasticity, no patients formed NAbs during the main period or extension phase of the trial [90]. As noted previously, patients with previous BoNT-A SNR have been restarted on incobotulinumtoxinA with sustained clinical response [43], and no patients in the TOWER study examining escalating incobotulinumtoxinA doses for spasticity developed NAbs by MHDA [72]. Even trials that have examined flexible dosing of incobotulinumtoxinA at intervals shorter than three months as per symptom-based assessment have noted no new NAb formation [22,27,91,92]. In a cross-sectional analysis of antibody prevalence across indications and BoNT-A formulations, no antibodies were found in patients treated with incobotulinumtoxinA, despite higher than expected overall antibody prevalence [51]. However, the overall shorter duration of incobotulinumtoxinA used compared to older formulations was noted as a confounding factor.

## 9. Immunogenicity and Duration of Treatment

Overall, immunogenicity rates of the BoNT-A formulations (excluding the original batch of onabotulinumtoxinA) have been low, with meta-analyses estimating the prevalence from 0.5% for incobotulinumtoxin, 1.5% for onabotulinumtoxinA, and 1.7% for abobotulinumtoxinA [19]. However, many of these studies are relatively short in duration, and evaluate for antibody formation after only a few injection cycles, or even after a single injection cycle. However, given the trend of higher NAb formation in indications with higher BoNT doses, concern has been raised for higher prevalence of NAbs as cumulative doses rise over longer courses of treatment. One study examined the rate of partial SNR over time in 660 patients with CD [93]. After defining partial SNR as a systematic reduction in Tsui scores (a rating scale of CD severity) and decline in patient-reported efficacy, they found that the rate of partial SNR development remained fairly constant, even after four years. Interestingly though, the subgroup of patients who would eventually develop partial SNR had early significant changes in the standardized Tsui scores by their third injection cycle as compared to those who would continue to have clinical responsiveness [93].

In 2016, Hefter et al. [94] examined antibody prevalence in CD patients treated with different formulation of BoNT over a long duration who continued to respond clinically. They analyzed 212 patients with NAb testing results; these samples were first screened with ELISA, followed by confirmatory testing with MHDA. The average treatment duration of these patients was 11.7 years. NAbs were found by MHDA in 31 of the 212 patients (14.6%). A yearly incidence of NAb formation was calculated to be 1.26% based on treatment duration. CD severity had been assessed by Tsui scores, and over the entire sample, Tsui scores remained relatively flat. However, while all patients still had clinical response, patients with NAbs by MHDA had overall higher Tsui scores, higher doses of BoNT required, and longer durations of therapy. Despite these higher scores, the authors noted there was “no obvious, good clinical criterion clearly predicting the presence of NAbs” [94].

Calculations of incidences of NAbs formation assume a linear increase over time. A previous study has questioned both the timing of NAbs development and the risk of NAbs associated SNR with higher cumulative dosing of BoNT [3]. In this study, 27 patients with NAbs-associated SNR were analyzed: SNR developed between 3–4 injections and 11–12 injections, and development of SNR due to NAbs was rare after 14 injections. Other available studies on long-term use of BoNT have shown overall lower prevalence of NAbs than these more recent estimates. In a study examining 45 patients who had received BoNT injections for over 12 years the initial injections were performed between 1985 and 1989 with the more immunogenic original batch of onabotulinumtoxinA [95]. Of the 45 patients, six developed SNR and four of these patients were found to have NAbs by MPA [95], which would fall within previously established range of 5%–17% antibody prevalence range for the original formulation of onabotulinumtoxinA [18].

In another study, a longitudinal cohort of 100 patients injected first between 1989 and 1992 (primarily with abobotulinumtoxinA) were analyzed; 33 patients had stopped BoNT therapy, but only three were due to presumed SNR. Of these three patients, none had NAb by MHDA and NAb were not assessed in the other patients [87]. Another study examining abotulinumtoxinA in CD compared 303 clinically responsive patients to 162 patients who had dropped out of therapy. Only 17 of these 162 patients had SNR, and nine of the 17 patients had NAbs by MPA, MHDA, or EDB clinical testing, yielding a NAb prevalence of 2.5%. These patients with NAbs had 11 injections on average (ranging from 7–14), and all of the 303 clinically responsive patients had at least six injections [23].

## 10. Discussion

There has been a great deal of research dedicated to exploring immunogenicity of BoNT. While certain trends of data have become apparent, such as the higher risk of immunogenicity of older formulations of onabotulinumtoxinA containing higher ratios of inactive proteins, many questions remain.

**Figure 2:**
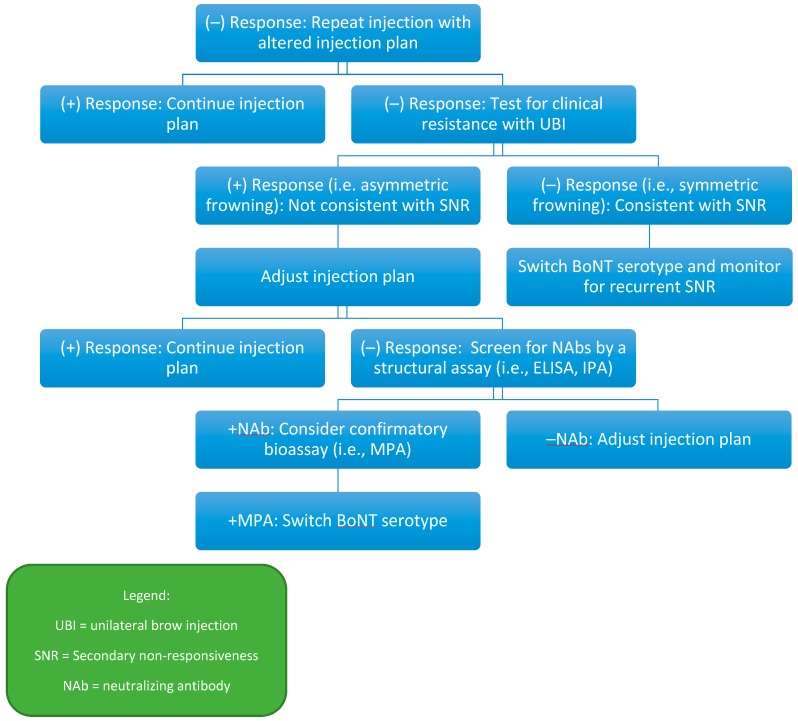
Proprosed secondary non-responsive (SNR) detection and management pathway.

**Figure 3:**
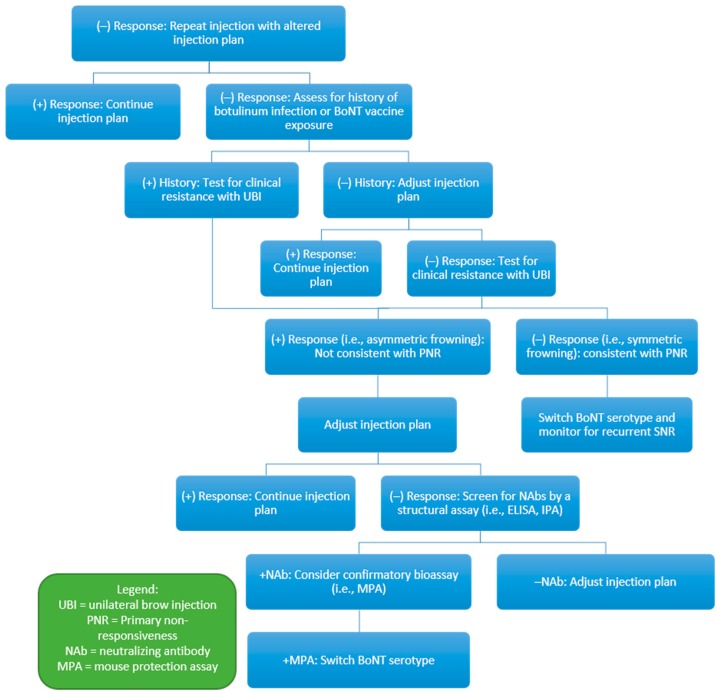
Proposed primary non-responsive (PNR) dectection and management pathway.

Perhaps the most notable uncertainty involves the exact relationship between NAbs and SNR. This uncertainty is partly due to a lack of quantitative, specific, and sensitive assays for NAbs. Furthermore, response to BoNT as determined by a subjective response or by application of clinical rating scales is difficult to ascertain reliably and objectively. In addition, any assessment of response must consider a placebo or nocebo effect, which makes the interpretation of possible correlation between antibodies and response difficult. While initial studies of MPA, considered by many as the “gold standard”, have reported a 100% specificity with this assay, its sensitivity has always been relatively low, less than 50% [59]. Even the specificity is difficult to interpret, as rare patients with NAbs determined by MPA continue to have a “clinical response”, although this may represent a placebo effect [18]. One study examined 214 patients with CD in a 10-week, open-label trial of onabotulinumtoxinA [96]. These patients were evaluated 6 weeks after injection by the physician global assessment scale (GAS) and the CD severity scale (CDSS). Patients who were deemed responders by these evaluations proceeded to the second, randomized controlled period of the trial, while non-responders were excluded. All patients had received at least two injections of onabotulinumtoxinA prior to enrollment. NAbs were detected by MPA in 32 of 191 available samples (17%) at baseline. Patients with and without NAbs at baseline exhibited similar statistically significant improvements in CDSS scores over 10 weeks, despite their antibody status [96]. This study is an example of how difficult it is to interpret antibody status and clinical response. For now, clinical resistance tests can be initially used in the evaluation of suspected SNR (Figure 2) and PNR (Figure 3) before proceeding to additional structural assays or bioassays.

The MHDA has been found to have low sensitivity: Analyses of patients with SNR have shown NAbs prevalence of only 53.5% [19] and 44.5% [20], respectively in two different studies. At the same time, the lower detection limit has raised concerns for increased rates of false positives [7]. In one meta-analysis, 3.5% of patients with continued clinical response had NAbs by MHDA [19]. However, studies looking only at clinically responsive patients have found an even higher prevalence of NAbs by MHDA despite continued clinical response [51,94]. Adding to the confusion, patients undergoing rimabotulinumtoxinB injections in particular appear to have notably high rates of NAb development, yet high proportions of these NAb-positive patients continue to have a clinical response, and, conversely, patients with SNR often have no NAbs [89]. In that same study, higher prevalence of NAbs were noted in patients with higher doses of rimabotulinumtoxinB.

There are many unmet needs that must be addressed in order to optimize outcomes related to BoNT treatments. In addition to developing new and better formulations of BoNT, we need clear definitions of clinical response and SNR. Novel, cost-effective structural assays are needed that measure titers of NAbs with high predictive values. With wider access to these assays, patients undergoing BoNT may be monitored for emergence of NAbs so that appropriate measures can be taken to prevent SNR.

## 11. Conclusions

BoNT has been a safe and effective treatment option for a variety of neurologic and non-neurologic conditions. While formation of NAbs continues to be a concern, several strategies can be used to mitigate against the development of immunoresistance. These include development of formulations of products that are associated with minimal protein load, avoidance of adjuvants, safe inter-injection interval, and the use of the smallest dose that provides optimal clinical response and does not compromise potential benefits. There is an urgent need to transition from bioassays to highly sensitive and specific structural assays that are inexpensive and readily accessible for long-term monitoring. Once these assays are available, they should be critically evaluated in longitudinal studies to define and clarify the relationship between NAbs and SNR. Better understanding of the immunologic mechanisms involved in the development of NAbs will undoubtedly lead to strategies to prevent or treat BoNT-related immunoresistance.

## Figures and Tables

**Table 1:** Assays to detect neutralizing antibodies.

Mouse Protection Assay (MPA)
Mouse Hemidiaphragm Assay (MHDA)
Immunoprecipitation Assay (IPA)
Western Blot Assay (WBA)
Synaptosome Inhibition Assay (SIA)
Enzyme-Linked Immunosorbent Assay (ELISA)
Sternocleidomastoid test (SCM)
Electrical stimulation of injected muscle (EDB)
Other assays (e.g., sudomotor, ninhydrin sweat test)
Clinical tests (UBI, FTAT)

**Table 2:** Clinical resistance tests.

Test Name	Injection Site	Required Tools	Clinically Responsive Result
UBI	Medial eyebrow	None	Asymmetric frowning
FTAT	Frontalis	None	Asymmetric forehead wrinkling
EDB	Extensor digitorum brevis	EMG	>50% decrease in EDB CMAP
SCM	Sternocleidomastoid	EMG	Maximal contraction reduction % >2 SD below mean control reduction %
NST	Hypothenar eminence	Ninhydrin solution	Decreased anhidrotic area

**Table 3:** Botulinum toxin formulations.

BoNT Formulation	Trade Names	FDA-Approved Indications	Estimated Equivalent Dose (to ONA)	Immunogenicity Ranges	Notes
Ona (OLD)	Botox^®^	Not in use	1	5–17% [18]	
Ona (New)	Botox^®^	CD, AH, BSP, ULS, OAB, CM, strabismus [8]	1	0.2% [60]–3.6% [82]	Reduced amounts of inactive protein compared to older formulation
Abo	Dysport^®^	CD, GL, ULS (adults), LLS (children) [77]	2.5	0.9% [83]–3.6% [77]	
Inco	Xeomin^®^	CD, BSP, GL, USL, sialorrhea [84]	1	0% [51]–0.5% [19]	NAPs removed
Rima	Myobloc^®^ (USA), NeuroBloc^®^	CD [85]	50	18% [85]–42.4% [19]	B serotype, stronger autonomic effects

Legend: Ona = onabotulinumtoxinA, Abo = abobotulinumtoxinA, Iinc = incobotulinumtoxinA, Rima = rimabotulinumtoxinB, CD = cervical dystonia, AH = axillary hyperhidrosis, BSP = blepharospasm, ULS = upper-limb spasticity, LLS = lower-limb spasticity, OAB = overactive bladder, CM = chronic migraine, GL = glabellar lines, NAPs = non-toxic accessory protein.

## References

[1] Jankovic J. (2017). Botulinum Toxin: State of the Art. Mov. Disord..

[2] Jankovic J., Orman J. (1987). Botulinum A Toxin for Cranial-Cervical Dystonia: A Double-Blind, Placebo-Controlled Study. Neurology.

[3] Dressler D. (2004). Clinical Presentation and Management of Antibody-Induced Failure of Botulinum Toxin Therapy. Mov. Disord..

[4] Hsiung G.Y.R., Das S.K., Ranawaya R., Lafontaine A.L., Suchowersky O. (2002). Long-Term Efficacy of Botulinum Toxin A in Treatment of Various Movement Disorders over a 10-Year Period. Mov. Disord..

[5] Ramirez-Castaneda J., Jankovic J. (2013). Long-Term Efficacy and Safety of Botulinum Toxin Injections in Dystonia. Toxins (Basel).

[6] Pittman P.R., Hack D., Mangiafico J., Gibbs P., McKee K.T., Friedlander A.M., Sjogren M.H. (2002). Antibody Response to a Delayed Booster Dose of Anthrax Vaccine and Botulinum Toxoid. Vaccine.

[7] Naumann M., Boo L.M., Ackerman A.H., Gallagher C.J. (2013). Immunogenicity of Botulinum Toxins. J. Neural Transm..

[8] (2019). BOTOX® [Package Insert].

[9] Trindade De Almeida A.R., Secco L.C., Carruthers A. (2011). Handling Botulinum Toxins: An Updated Literature Review. Dermatol. Surg..

[10] Gartlan M.G., Hoffman H.T. (1993). Crystalline Preparation of Botulinum Toxin Type A (Botox): Degradation in Potency with Storage. Otolaryngol. Head. Neck Surg..

[11] Albanese A., Bhatia K., Bressman S.B., Delong M.R., Fahn S., Fung V.S.C., Hallett M., Jankovic J., Jinnah H.A., Klein C. (2013). Phenomenology and Classification of Dystonia: A Consensus Update. Mov. Disord..

[12] Pirazzini M., Carle S., Barth H., Rossetto O., Montecucco C. (2018). Primary Resistance of Human Patients to Botulinum Neurotoxins A and B. Ann. Clin. Transl. Neurol..

[13] Oshima M., Deitiker P., Hastings-Ison T., Aoki K.R., Graham H.K., Atassi M.Z. (2017). Antibody Responses to Botulinum Neurotoxin Type A of Toxin-Treated Spastic Equinus Children with Cerebral Palsy: A Randomized Clinical Trial Comparing Two Injection Schedules. J. Neuroimmunol..

[14] Atassi M.Z., Dolimbek B.Z., Jankovic J., Steward L.E., Aoki K.R. (2011). Regions of Botulinum Neurotoxin A Light Chain Recognized by Human Anti-Toxin Antibodies from Cervical Dystonia Patients Immunoresistant to Toxin Treatment. The Antigenic Structure of the Active Toxin Recognized by Human Antibodies. Immunobiology.

[15] Baizabal-Carvallo J.F., Jankovic J., Feld J. (2013). Flu-like Symptoms and Associated Immunological Response Following Therapy with Botulinum Toxins. Neurotox. Res..

[16] Jankovic J., Schwartz K. (1995). Response and Immunoresistance to Botulinum Toxin Injections. Neurology.

[17] Jinnah H.A., Goodmann E., Rosen A.R., Evatt M., Freeman A., Factor S. (2016). Botulinum Toxin Treatment Failures in Cervical Dystonia: Causes, Management, and Outcomes. J. Neurol..

[18] Brin M.F., Comella C.L., Jankovic J., Lai F., Naumann M., CD-017 BoNTA Study Group (2008). Long-Term Treatment with Botulinum Toxin Type A in Cervical Dystonia Has Low Immunogenicity by Mouse Protection Assay. Mov. Disord..

[19] Fabbri M., Leodori G., Fernandes R.M., Bhidayasiri R., Marti M.J., Colosimo C., Ferreira J.J. (2016). Neutralizing Antibody and Botulinum Toxin Therapy: A Systematic Review and Meta-Analysis. Neurotox. Res..

[20] Lange O., Bigalke H., Dengler R., Wegner F., DeGroot M., Wohlfarth K. (2009). Neutralizing Antibodies and Secondary Therapy Failure after Treatment with Botulinum Toxin Type A: Much Ado about Nothing?. Clin. Neuropharmacol..

[21] Atassi M.Z., Dolimbek B.Z., Jankovic J., Steward L.E., Aoki K.R. (2008). Molecular Recognition of Botulinum Neurotoxin B Heavy Chain by Human Antibodies from Cervical Dystonia Patients That Develop Immunoresistance to Toxin Treatment. Mol. Immunol..

[22] Dressler D., Saberi F.A. (2017). Safety of Botulinum Toxin Short Interval Therapy Using Incobotulinumtoxin A. J. Neural Transm..

[23] Kessler K.R., Skutta M., Benecke R., German Dystonia Study Group (1999). Long-Term Treatment of Cervical Dystonia with Botulinum Toxin A: Efficacy, Safety, and Antibody Frequency. J. Neurol..

[24] Dressler D., Bigalke H. (2017). Immunological Aspects of Botulinum Toxin Therapy. Expert Rev. Neurother..

[25] Ojo O.O., Fernandez H.H. (2015). Is It Time for Flexibility in Botulinum Inter-Injection Intervals?. Toxicon.

[26] Sethi K.D., Rodriguez R., Olayinka B. (2012). Satisfaction with Botulinum Toxin Treatment: A Cross-Sectional Survey of Patients with Cervical Dystonia. J. Med. Econ..

[27] Evidente V.G.H., Fernandez H.H., LeDoux M.S., Brashear A., Grafe S., Hanschmann A., Comella C.L. (2013). A Randomized, Double-Blind Study of Repeated IncobotulinumtoxinA (Xeomin(^®^)) in Cervical Dystonia. J. Neural Transm..

[28] Truong D.D., Gollomp S.M., Jankovic J., LeWitt P.A., Marx M., Hanschmann A., Fernandez H.H. (2013). Xeomin US Blepharospasm Study Group. Sustained Efficacy and Safety of Repeated IncobotulinumtoxinA (Xeomin(^®^)) Injections in Blepharospasm. J. Neural Transm..

[29] Kukreja R.V., Singh B.R. (2007). Comparative Role of Neurotoxin-Associated Proteins in the Structural Stability and Endopeptidase Activity of Botulinum Neurotoxin Complex Types A and E. Biochemistry.

[30] Dong M., Yeh F., Tepp W.H., Dean C., Johnson E.A., Janz R., Chapman E.R. (2006). SV2 Is the Protein Receptor for Botulinum Neurotoxin A. Science.

[31] Pirazzini M., Rossetto O., Eleopra R., Montecucco C. (2017). Botulinum Neurotoxins: Biology, Pharmacology, and Toxicology. Pharmacol. Rev..

[32] Kukreja R., Chang T.W., Cai S., Lindo P., Riding S., Zhou Y., Ravichandran E., Singh B.R. (2009). Immunological Characterization of the Subunits of Type A Botulinum Neurotoxin and Different Components of Its Associated Proteins. Toxicon.

[33] Frevert J., Dressler D. (2010). Complexing Proteins in Botulinum Toxin Type A Drugs: A Help or a Hindrance?. Biologics.

[34] Jankovic J., Truong D., Patel A.T., Brashear A., Evatt M., Rubio R.G., Oh C.K., Snyder D., Shears G., Comella C. (2018). Injectable DaxibotulinumtoxinA in Cervical Dystonia: A Phase 2 Dose-Escalation Multicenter Study. Mov. Disord. Clin. Pract..

[35] Dirk D., Mander G.J., Klaus F. (2008). Equivalent Potency of Xeomin^®^ and BOTOX^®^. Toxicon.

[36] Ranoux D., Gury C., Fondarai J., Mas J.L., Zuber M. (2002). Respective Potencies of Botox and Dysport: A Double Blind, Randomised, Crossover Study in Cervical Dystonia. J. Neurol. Neurosurg. Psychiatry.

[37] Comella C.L., Jankovic J., Shannon K.M., Tsui J., Swenson M., Leurgans S., Fan W., Dystonia Study Group (2005). Comparison of Botulinum Toxin Serotypes A and B for the Treatment of Cervical Dystonia. Neurology.

[38] Dressler D., Benecke R. (2003). Autonomic Side Effects of Botulinum Toxin Type B Treatment of Cervical Dystonia and Hyperhidrosis. Eur. Neurol..

[39] Carruthers J., Solish N., Humphrey S., Rosen N., Muhn C., Bertucci V., Swift A., Metelitsa A., Rubio R.G., Waugh J. (2017). Injectable DaxibotulinumtoxinA for the Treatment of Glabellar Lines: A Phase 2, Randomized, Dose-Ranging, Double-Blind, Multicenter Comparison With OnabotulinumtoxinA and Placebo. Dermatol. Surg..

[40] Oshima M., Deitiker P., Jankovic J., Atassi M.Z. (2018). The Regions on the Light Chain of Botulinum Neurotoxin Type A Recognized by T Cells from Toxin-Treated Cervical Dystonia Patients. The Complete Human T-Cell Recognition Map of the Toxin Molecule. Immunol. Investig..

[41] Sankhla C., Jankovic J., Duane D. (1998). Variability of the Immunologic and Clinical Response in Dystonic Patients Immunoresistant to Botulinum Toxin Injections. Mov. Disord..

[42] Dressler D., Bigalke H. (2002). Botulinum Toxin Antibody Type A Titres after Cessation of Botulinum Toxin Therapy. Mov. Disord..

[43] Dressler D., Pan L., Adib Saberi F. (2018). Antibody-Induced Failure of Botulinum Toxin Therapy: Re-Start with Low-Antigenicity Drugs Offers a New Treatment Opportunity. J. Neural Transm..

[44] Dressler D., Münchau A., Bhatia K.P., Quinn N.P., Bigalke H. (2002). Antibody-Induced Botulinum Toxin Therapy Failure: Can It Be Overcome by Increased Botulinum Toxin Doses?. Eur. Neurol..

[45] Dressler D., Zettl U., Benecke R., Bigalke H. (2000). Can Intravenous Immunoglobulin Improve Antibody-Mediated Botulinum Toxin Therapy Failure?. Mov. Disord..

[46] Dolimbek B.Z., Steward L.E., Aoki K.R., Atassi M.Z. (2012). Location of the Synaptosome-Binding Regions on Botulinum Neurotoxin B. Biochemistry.

[47] Chen R., Karp B.I., Hallett M. (1998). Botulinum Toxin Type F for Treatment of Dystonia: Long-Term Experience. Neurology.

[48] Joshi S.G., Elias M., Singh A., Al-Saleem F.H., Ancharski D., Nasser Z., Takahashi T., Simpson L.L. (2011). Modulation of Botulinum Toxin-Induced Changes in Neuromuscular Function with Antibodies Directed against Recombinant Polypeptides or Fragments. Neuroscience.

[49] Yoon S., Kurnasov O., Natarajan V., Hong M., Gudkov A.V., Osterman A.L., Wilson I.A. (2012). Structural Basis of TLR5-Flagellin Recognition and Signaling. Science.

[50] Lee J.C., Yokota K., Arimitsu H., Hwang H.J., Sakaguchi Y., Cui J., Takeshi K., Watanabe T., Ohyama T., Oguma K. (2005). Production of Anti-Neurotoxin Antibody Is Enhanced by Two Subcomponents, HA1 and HA3b, of Clostridium Botulinum Type B 16S Toxin-Haemagglutinin. Microbiology.

[51] Albrecht P., Jansen A., Lee J.I., Moll M., Ringelstein M., Rosenthal D., Bigalke H., Aktas O., Hartung H.P., Hefter H. (2019). High Prevalence of Neutralizing Antibodies after Long-Term Botulinum Neurotoxin Therapy. Neurology.

[52] Aoki K.R., Guyer B. (2001). Botulinum Toxin Type A and Other Botulinum Toxin Serotypes: A Comparative Review of Biochemical and Pharmacological Actions. Eur. J. Neurol..

[53] Dressler D., Hallett M. (2006). Immunological Aspects of Botox, Dysport and Myobloc/NeuroBloc. Eur. J. Neurol..

[54] Jankovic J., Vuong K.D., Ahsan J. (2003). Comparison of Efficacy and Immunogenicity of Original versus Current Botulinum Toxin in Cervical Dystonia. Neurology.

[55] Callaway J.E. (2004). Botulinum Toxin Type B (Myobloc): Pharmacology and Biochemistry. Clin. Dermatol..

[56] Jankovic J., Hunter C., Dolimbek B.Z., Dolimbek G.S., Adler C.H., Brashear A., Comella C.L., Gordon M., Riley D.E., Sethi K. (2006). Clinico-Immunologic Aspects of Botulinum Toxin Type B Treatment of Cervical Dystonia. Neurology.

[57] Gottlieb S. (2008). Biosimilars: Policy, Clinical, and Regulatory Considerations. Am. J. Health. Syst. Pharm..

[58] Hanna P.A., Jankovic J., Vincent A. (1999). Comparison of Mouse Bioassay and Immunoprecipitation Assay for Botulinum Toxin Antibodies. J. Neurol. Neurosurg. Psychiatry.

[59] Hanna P.A., Jankovic J. (1998). Mouse Bioassay versus Western Blot Assay for Botulinum Toxin Antibodies: Correlation with Clinical Response. Neurology.

[60] Naumann M., Carruthers A., Carruthers J., Aurora S.K., Zafonte R., Abu-Shakra S., Boodhoo T., Miller-Messana M.A., Demos G., James L. (2010). Meta-Analysis of Neutralizing Antibody Conversion with OnabotulinumtoxinA (BOTOX^®^) across Multiple Indications. Mov. Disord..

[61] Dressler D., Dirnberger G., Bhatia K.P., Irmer A., Quinn N.P., Bigalke H., Marsden C.D. (2000). Botulinum Toxin Antibody Testing: Comparison between the Mouse Protection Assay and the Mouse Lethality Assay. Mov. Disord..

[62] Göschel H., Wohlfarth K., Frevert J., Dengler R., Bigalke H. (1997). Botulinum A Toxin Therapy: Neutralizing and Nonneutralizing Antibodies—Therapeutic Consequences. Exp. Neurol..

[63] Sesardic D., Jones R.G.A., Leung T., Alsop T., Tierney R. (2004). Detection of Antibodies against Botulinum Toxins. Mov. Disord..

[64] Dressler D., Gessler F., Tacik P., Bigalke H. (2014). An Enzyme-Linked Immunosorbent Assay for Detection of Botulinum Toxin-Antibodies. Mov. Disord..

[65] Gordon P.H., Gooch C.L., Greene P.E. (2002). Extensor Digitorum Brevis Test and Resistance to Botulinum Toxin Type A. Muscle Nerve.

[66] Cordivari C., Misra V.P., Vincent A., Catania S., Bhatia K.P., Lees A.J. (2006). Secondary Nonresponsiveness to Botulinum Toxin A in Cervical Dystonia: The Role of Electromyogram-Guided Injections, Botulinum Toxin A Antibody Assay, and the Extensor Digitorum Brevis Test. Mov. Disord..

[67] Dressler D., Bigalke H., Rothwell J.C. (2000). The Sternocleidomastoid Test: An in Vivo Assay to Investigate Botulinum Toxin Antibody Formation in Humans. J. Neurol..

[68] Voller B., Moraru E., Auff E., Benesch M., Poewe W., Wissel J., Müller J., Entner T., Bigalke H., Schnider P. (2004). Ninhydrin Sweat Test: A Simple Method for Detecting Antibodies Neutralizing Botulinum Toxin Type A. Mov. Disord..

[69] Kranz G.S., Sycha T., Voller B., Kranz G.S., Schnider P., Auff E. (2008). Neutralizing Antibodies in Dystonic Patients Who Still Respond Well to Botulinum Toxin Type A. Neurology.

[70] Elovic E.P., Brashear A., Kaelin D., Liu J., Millis S.R., Barron R., Turkel C. (2008). Repeated Treatments with Botulinum Toxin Type a Produce Sustained Decreases in the Limitations Associated with Focal Upper-Limb Poststroke Spasticity for Caregivers and Patients. Arch. Phys. Med. Rehabil..

[71] Bakheit A.M.O., Fedorova N.V., Skoromets A.A., Timerbaeva S.L., Bhakta B.B., Coxon L. (2004). The Beneficial Antispasticity Effect of Botulinum Toxin Type A Is Maintained after Repeated Treatment Cycles. J. Neurol. Neurosurg. Psychiatry.

[72] Wissel J., Bensmail D., Ferreira J.J., Molteni F., Satkunam L., Moraleda S., Rekand T., McGuire J., Scheschonka A., Flatau-Baqué B. (2017). Safety and Efficacy of IncobotulinumtoxinA Doses up to 800 U in Limb Spasticity: The TOWER Study. Neurology.

[73] Cruz F., Herschorn S., Aliotta P., Brin M., Thompson C., Lam W., Daniell G., Heesakkers J., Haag-Molkenteller C. (2011). Efficacy and Safety of OnabotulinumtoxinA in Patients with Urinary Incontinence Due to Neurogenic Detrusor Overactivity: A Randomised, Double-Blind, Placebo-Controlled Trial. Eur. Urol..

[74] Schulte-Baukloh H., Bigalke H., Heine G., Pape D., Stuerzebecher B., Miller K., Knispel H.H. (2007). Antibodies against Botulinum Neurotoxin Type A as a Cause of Treatment Failure after the First Detrusor Injection. Urology.

[75] Schulte-Baukloh H., Bigalke H., Miller K., Heine G., Pape D., Lehmann J., Knispel H.H. (2008). Botulinum Neurotoxin Type A in Urology: Antibodies as a Cause of Therapy Failure. Int. J. Urol..

[76] Kawashima M., Harii K. (2009). An Open-Label, Randomized, 64-Week Study Repeating 10- and 20-U Doses of Botulinum Toxin Type A for Treatment of Glabellar Lines in Japanese Subjects. Int. J. Dermatol..

[77] (2016). DYSPORT^®^ [Package Insert].

[78] Monheit G.D., Cohen J.L., Reloxin Investigational Group (2009). Long-Term Safety of Repeated Administrations of a New Formulation of Botulinum Toxin Type A in the Treatment of Glabellar Lines: Interim Analysis from an Open-Label Extension Study. J. Am. Acad. Dermatol..

[79] Moy R., Maas C., Monheit G., Huber M.B., Reloxin Investigational Group (2009). Long-Term Safety and Efficacy of a New Botulinum Toxin Type A in Treating Glabellar Lines. Arch. Facial Plast. Surg..

[80] Imhof M., Kühne U. (2011). A Phase III Study of IncobotulinumtoxinA in the Treatment of Glabellar Frown Lines. J. Clin. Aesthet. Dermatol..

[81] Glaser D.A., Pariser D.M., Hebert A.A., Landells I., Somogyi C., Weng E., Brin M.F., Beddingfield F. (2015). A Prospective, Nonrandomized, Open-Label Study of the Efficacy and Safety of OnabotulinumtoxinA in Adolescents with Primary Axillary Hyperhidrosis. Pediatr. Dermatol..

[82] Lacroix-Desmazes S., Mouly S., Popoff M.R., Colosimo C. (2017). Systematic Analysis of Botulinum Neurotoxin Type A Immunogenicity in Clinical Studies. Basal Ganglia.

[83] Truong D., Brodsky M., Lew M., Brashear A., Jankovic J., Molho E., Orlova O., Timerbaeva S., Global Dysport Cervical Dystonia Study Group (2010). Long-Term Efficacy and Safety of Botulinum Toxin Type A (Dysport) in Cervical Dystonia. Parkinsonism Relat. Disord..

[84] (2018). XEOMIN^®^ [Package Insert].

[85] (2009). MYOBLOC^®^ [Package Insert].

[86] Coleman C., Hubble J., Schwab J., Beffy J.L., Picaut P., Morte C. (2012). Immunoresistance in Cervical Dystonia Patients after Treatment with AbobotulinumtoxinA. Int. J. Neurosci..

[87] Haussermann P., Marczoch S., Klinger C., Landgrebe M., Conrad B., Ceballos-Baumann A. (2004). Long-Term Follow-up of Cervical Dystonia Patients Treated with Botulinum Toxin A. Mov. Disord..

[88] Field M., Splevins A., Picaut P., van der Schans M., Langenberg J., Noort D., Snyder D., Foster K. (2018). AbobotulinumtoxinA (Dysport^®^), OnabotulinumtoxinA (Botox^®^), and IncobotulinumtoxinA (Xeomin^®^) Neurotoxin Content and Potential Implications for Duration of Response in Patients. Toxins (Basel).

[89] Chinnapongse R.B., Lew M.F., Ferreira J.J., Gullo K.L., Nemeth P.R., Zhang Y. (2012). Immunogenicity and Long-Term Efficacy of Botulinum Toxin Type B in the Treatment of Cervical Dystonia: Report of 4 Prospective, Multicenter Trials. Clin. Neuropharmacol..

[90] Lamb Y.N., Scott L.J. (2016). IncobotulinumtoxinA: A Review in Upper Limb Spasticity. Drugs.

[91] Evidente V.G.H., Truong D., Jankovic J., Comella C.L., Grafe S., Hanschmann A. (2014). IncobotulinumtoxinA (Xeomin^®^) Injected for Blepharospasm or Cervical Dystonia According to Patient Needs Is Well Tolerated. J. Neurol. Sci..

[92] Dressler D., Paus S., Seitzinger A., Gebhardt B., Kupsch A. (2013). Long-Term Efficacy and Safety of IncobotulinumtoxinA Injections in Patients with Cervical Dystonia. J. Neurol. Neurosurg. Psychiatry.

[93] Hefter H., Spiess C., Rosenthal D. (2014). Very Early Reduction in Efficacy of Botulinum Toxin Therapy for Cervical Dystonia in Patients with Subsequent Secondary Treatment Failure: A Retrospective Analysis. J. Neural Transm..

[94] Hefter H., Rosenthal D., Moll M. (2016). High Botulinum Toxin-Neutralizing Antibody Prevalence Under Long-Term Cervical Dystonia Treatment. Mov. Disord. Clin. Pract..

[95] Mejia N.I., Vuong K.D., Jankovic J. (2005). Long-Term Botulinum Toxin Efficacy, Safety, and Immunogenicity. Mov. Disord..

[96] Charles D., Brashear A., Hauser R.A., Li H.I., Boo L.M., Brin M.F., CD 140 Study Group (2012). Efficacy, Tolerability, and Immunogenicity of Onabotulinumtoxina in a Randomized, Double-Blind, Placebo-Controlled Trial for Cervical Dystonia. Clin. Neuropharmacol..

